# Reservoir based spiking models for univariate Time Series Classification

**DOI:** 10.3389/fncom.2023.1148284

**Published:** 2023-06-08

**Authors:** Ramashish Gaurav, Terrence C. Stewart, Yang Yi

**Affiliations:** ^1^Department of Electrical and Computer Engineering, Virginia Tech, Blacksburg, VA, United States; ^2^University of Waterloo Collaboration Centre, National Research Council of Canada, Waterloo, ON, Canada

**Keywords:** Legendre Memory Units, Time Series Classification (TCS), Spiking Neural Network (SNN), Surrogate Gradient Descent, Loihi, Reservoir Computing (RC)

## Abstract

A variety of advanced machine learning and deep learning algorithms achieve state-of-the-art performance on various temporal processing tasks. However, these methods are heavily energy inefficient—they run mainly on the power hungry CPUs and GPUs. Computing with Spiking Networks, on the other hand, has shown to be energy efficient on specialized neuromorphic hardware, e.g., Loihi, TrueNorth, SpiNNaker, etc. In this work, we present two architectures of spiking models, inspired from the theory of Reservoir Computing and Legendre Memory Units, for the Time Series Classification (TSC) task. Our first spiking architecture is closer to the general Reservoir Computing architecture and we successfully deploy it on Loihi; the second spiking architecture differs from the first by the inclusion of non-linearity in the readout layer. Our second model (trained with Surrogate Gradient Descent method) shows that non-linear decoding of the linearly extracted temporal features through spiking neurons not only achieves promising results, but also offers low computation-overhead by significantly reducing the number of neurons compared to the popular LSM based models—more than 40x reduction with respect to the recent spiking model we compare with. We experiment on five TSC datasets and achieve new SoTA spiking results (—as much as 28.607% accuracy improvement on one of the datasets), thereby showing the potential of our models to address the TSC tasks in a green energy-efficient manner. In addition, we also do energy profiling and comparison on Loihi and CPU to support our claims.

## 1. Introduction

Almost all of the signals around us are intrinsically temporal (e.g., audio/speech, sensor signals, etc.) or have a temporal component to it (e.g., video/vision signals, etc.). Machine Learning (ML) and Deep Learning (DL) algorithms, no doubt, have catered well to the growing processing needs of temporal datasets (Pan et al., 2022)—with respect to scalability, variety, and robustness, etc. However, one evident drawback of the traditional ML/DL algorithms [e.g., LSTM (Hochreiter and Schmidhuber, 1997), HIVE-COTE (Lines et al., 2018), ResNet (He et al., 2016), etc.] is their energy *inefficiency* when deployed on general purpose CPUs/GPUs/FPGAs. This energy-intensive characteristic of the conventional/DL Time-Series models makes them poorly suited to the energy constrained devices/applications. On the contrary, Spiking Neural Networks (SNNs), the next generation of neural networks is gaining prominence due to their promise of low power and low latency AI when deployed on specialized neuromorphic hardware, e.g., Intel's Loihi, IBM's TrueNorth, SpiNNaker, etc.

For simpler Time-Series datasets (e.g., signals from sensors), Reservoir Computing (Lukoševičius and Jaeger, 2009) based models are not only fast but also perform at par with complex time-series models (Bianchi et al., 2020), e.g., LSTM-FCN (Karim et al., 2017). In the Reservoir Computing (RC) paradigm, the input units are connected to a reservoir of randomly interconnected non-linear internal units with static weights, which is then further connected to a single trainable linear readout layer. The reservoir of internal units generate a high-dimensional temporal feature-map (i.e., extract temporal features from the input) on which the linear readout layer performs a linear transformation. Note that the connection weights of the readout layer are trained using regression methods.

In this work, the primary problem we aim to address is: “*How do we leverage the computational efficiency of Reservoir Computing methods to develop spiking architectures for Time Series Classification (TSC)?—which are not only energy efficient but also high performing*.” To this end, we present two spiking network architectures for the Time Series Classification (TSC) task of univariate signals. We develop our spiking models with neuromorphic hardware compatibility in mind, especially deployment on Intel's Loihi boards.

Echo State Network (ESN) (Jaeger, 2001), Liquid State Machine (LSM) (Maass et al., 2002), and Delayed Feedback Reservoir (DFR) (Appeltant et al., 2011; Bai and Yi, 2018) are widely studied/used RC models. Since the ESNs are non-spiking, they aren't suitable for neuromorphic hardware deployment. DFRs on the other hand are spiking networks, but to the best of our knowledge, they haven't been evaluated on any neuromorphic hardware. Furthermore, DFRs are much simpler architectures that merely keep track of temporally shifted inputs (Nowshin et al., 2020); they do not extract temporal features from the input signal. LSMs too are composed of spiking neurons and have been recently deployed on Loihi (Shenoy Renjal, 2019) (authors do a proof-of-concept demonstration with experiment on a small subset of a speech dataset) and on the SpiNNaker-103 board (Patiño-Saucedo et al., 2022). However, the dedicated circuits for synapses and spiking neurons in Loihi make it more efficient for processing low dimensional input signals than Spinnaker2 with general purpose ARM processors (Yan et al., 2021). Note that SpiNNaker-103 is less energy efficient than SpiNNaker2 (Yan et al., 2021) (and plausibly Loihi), since it is an older generation SpiNNaker1 board and does not have the MAC array. Note that apart from the RC based spiking models, a few DL inspired SNNs for TSC also exist; however, either they are too complex models which employ convolutions to extract features (Dominguez-Morales et al., 2018; Gautam and Singh, 2020) or use a relatively high number of trainable parameters in their architectures [more than 120000 in Fang et al. (2020)—although, for multivariate TSC]. This makes them less desirable for the resource constrained Edge/IoT devices. We next briefly introduce the Legendre Memory Unit (Voelker et al., 2019) which has the characteristics of a RC model.

Legendre Memory Unit (LMU) (Voelker et al., 2019) is a novel type of memory cell for RNNs that is based on the Delay Network (Voelker and Eliasmith, 2018); LMU has already shown promise in a wide range of AI tasks (Blouw et al., 2020; Chilkuri and Eliasmith, 2021; Chilkuri et al., 2021). Voelker et al. (2019) mention that the LMU memory cells (or the Delay Networks) can be implemented using spiking neurons; we refer to this implementation here as the Legendre Delay Network (LDN)—more details in Voelker and Eliasmith (2018). The LDN is based on the Linear Time-Invariant (LTI) system,


(1a) $$\dot{x}(t)=Ax(t)+Bu(t)$$

(1b) y(t)=Cx(t)+Du(t)

where **u**(*t*), **x**(*t*), $\dot{x}(t)$, and **y**(*t*) are the system's input, system's state, its time derivative, and system's output, respectively; *A, B, C*, and *D* are the time-invariant matrices defining the LTI system. Note that the spiking model of the LDN can be implemented using the principles of Neural Engineering Framework (NEF) (Eliasmith and Anderson, 2003; Stewart, 2012). Also note that NEF/LDN performs better than LSMs for implementing time delays (Voelker, 2019). We base both of our spiking models for TSC on LDN, and find that they indeed perform better than LSMs.

While one of our proposed spiking models follows the architecture of conventional RC models, our other model introduces non-linearity in the readout layer, such that the readout layer post the reservoir has a hidden layer of spiking neurons before the output layer. Direct training of SNNs is non-trivial; conventional back-propagation algorithm to train ANNs is not applicable to SNNs natively. This is primarily due to the non-differentiability of spikes while calculating the gradient of the loss function, as well as, due to the inherent temporality of SNNs. Although, a few methods exist to train SNNs (Pfeiffer and Pfeil, 2018), with the ANN-to-SNN conversion being extensively studied (Rueckauer and Liu, 2018; Bu et al., 2021; Li et al., 2021; Datta and Beerel, 2022; Gaurav et al., 2022b), where an already trained ANN is converted to an SNN by replacing the rate neurons (e.g., ReLU) with spiking neurons (e.g., Integrate & Fire), along with the other required network modifications. However, the ANN-to-SNN conversion suffers with two apparent disadvantages—(1): it fails to leverage the inherent temporal dynamics of SNNs while training and (2): it rips off any opportunity to train a high performance SNN on existing neuromorphic hardware in an energy efficient manner. Direct training of SNNs (Lee et al., 2016; Wu et al., 2018, 2019; Neftci et al., 2019; Zheng et al., 2021) intends to address these two problems. At the heart of direct training (most recent works), lies the approximation of the spike derivative with a surrogate derivative, which enables the back-propagation of the error-gradients to the deeper layers. In our second model with non-linear readout layer, we use this Surrogate Gradient Descent (SurrGD) approach to train it; we provide more details later. Note that this work is an extension of our previous work (Gaurav et al., 2022a) (recently published) where we developed a Spiking Reservoir Computing (SRC) model and deployed it on Loihi—in one of the firsts. For the sake of completeness, we present the relevant details of our previous work here. Sections 2.3, 3.1.1, 3.2, 4.1, and 5.1 are reused from our previous work. We next lay down our major contributions [contributions from Gaurav et al. (2022a) are italicized, rest are novel to this work]:

1. We propose two novel spiking architectures for the TSC of univariate signals
  - *Spiking Legendre Reservoir Computing (SLRC)* model (note that SRC model is renamed to SLRC)
  - Legendre Spiking Neural Network (LSNN) that achieves SoTA spiking results on used datasets

*2. In one of the firsts, we deploy our SLRC model on Loihi and do inference*. Loihi has been gaining prominence within the neuromorphic community with timely updates to the hardware (Davies et al., 2018; Intel, 2021; Orchard et al., 2021). For efficiency reasons stated above and up-to-date hardware, we aimed our work toward Loihi deployment.
3. The LSNN model is highly resource efficient—uses as low as 120 (or lesser) number of spiking neurons (depends on a hyper-parameter *d*—explained later), and is more than 40x resource efficient than the compared LSM based model. It is also more than 30x energy efficient than its non-spiking counterpart.
4. We support our claims with exhaustive experiments on five TSC datasets, along with energy profiling on CPU and Intel's Loihi-1 board.

We organize our paper as follows. In Section 2, we describe the theory behind our proposed spiking models: SLRC and LSNN, followed by the Section 3 where we explain the training and evaluation details of our experiments. We then present a detailed analysis of our results in the Section 4, followed by a discussion on our models and energy consumption in Section 5. We then finally conclude this work and lay down the future work prospects in the Section 6.

## 2. Methods

In this section, we describe the theoretical underpinnings of our proposed spiking models. We start with a brief explanation of the LDN [proposed by Voelker and Eliasmith (2018) with detailed explanations in Voelker (2019)], followed by the specifics of the Surrogate Gradient Descent (SurrGD) method. We then describe the architecture of our two proposed spiking univariate-TSC models—the Spiking Legendre Reservoir Computing (SLRC) model and the Legendre Spiking Neural Network (LSNN) model. Note that both the models are based on the LDN; in the SLRC model, the LDN is implemented with spiking neurons (using the principles of NEF), whereas in the LSNN model, the LDN is implemented with regular matrix operations. We use the Nengo (Stewart, 2012; Bekolay et al., 2014) and PyTorch (Paszke et al., 2019) libraries to build the SLRC and LSNN models, respectively. Henceforth, all the instances of neuron imply Integrate & Fire (IF) spiking neuron, unless otherwise stated.

### 2.1. Legendre delay network (LDN)

LDN is a type of an RNN (or a dynamical system) which implements a continuous-time delay of an input signal. We can mathematically represent a delay of an input signal **u**(*t*) as follows:


(2) y(t)=u(t−θ)

where θ ∈ ℝ^+^ is the time-seconds by which the input **u**(*t*) is delayed, and **y**(*t*) is the delayed output (by design, θ is limited to the duration of **u**(*t*)). To implement a system where the input is **u**(*t*), and the output is **y**(*t*), a straightforward way is to determine the Transfer function of such a system where it maps **u**(*t*) to the delayed output **u**(*t*−θ). Transfer functions are written as a ratio of *terms* in the complex variable *s*; the *terms* are the Laplace transforms of the input **u**(*t*) and the output **y**(*t*). To decompose **y**(*t*) (in Equation 2) as a function of **u**(*t*) and θ (to facilitate the calculation of the Transfer function), we observe the following.

An important property of the Impulse function (i.e., the Dirac's δ(*t*) function) is that when a shifted Impulse, i.e., δ(*t*−θ) is convolved with another time domain function, e.g., *f*(*t*), it sifts out the value of the function *f* at time *t*−θ; also called as the *sifting* property. That is:


(3) $$\begin{array}{l} f\left(t\right)*\delta \left(t-\theta \right)=f\left(t-\theta \right) \end{array}$$

where * is the convolution operator. Therefore, we can rewrite the Equation (2) as a convolution of the input signal **u**(*t*) and δ(*t*−θ) as follows (note, here *f*(*t*) is replaced by **u**(*t*), and δ_θ_(*t*) is short for δ(*t*−θ)):


(4a) $$y(t)=u(t)*\delta_{\theta }(t)$$

(4b) =u(t−θ)

Now that we have decomposed **y**(*t*) into **u**(*t*) and δ_θ_(*t*) in the Equation (4a), we can further simplify the Equation (4a) by taking its Laplace transform, i.e., $L\left[\text{y}\left(t\right)\right]=L\left[\text{u}\left(t\right)*\delta_{\theta }\left(t\right)\right]=L\left[\text{u}\left(t\right)\right]L\left[\delta_{\theta }\left(t\right)\right]$ (convolution in time domain becomes multiplication in Laplace domain); we can rearrange it as follows:


(5a) $$\begin{array}{ll} \frac{L\left[\text{y}\left(t\right)\right]}{L\left[\text{u}\left(t\right)\right]} & =L\left[\delta_{\theta }\left(t\right)\right] \end{array}$$

(5b) $$\Rightarrow \frac{Y(s)}{U(s)}=e^{-\theta s}$$

(5c) $$\Rightarrow ℱ(s)=e^{-\theta s}$$

where *e*^−θ*s*^ in Equation (5b) is the Laplace transform of the shifted Impulse function δ_θ_(*t*), and F(s) is the Transfer function of the system which implements a delay of θ time-seconds. Note that a Transfer function can be converted to an LTI state-space model (Equation 1) if and only if it can be written as a proper ratio of finite order polynomials in *s* (Brogan, 1991); Voelker presents a detailed derivation for the same in Voelker (2019), thereby obtaining the state space matrices *A* and *B* (in Equation 1a) as follows (we do not use Equation 1b in our models):


(6a) $$A={\left[a\right]}_{i,j},\;a_{i,j}=(2i+1)\{\begin{array}{l} -1\;i<j\\ {\left(-1\right)}^{i-j+1}\;i\geq j \end{array}$$

(6b) $$\begin{array}{l} B={\left[b\right]}_{i},\;b_{i}=\left(2i+1\right){\left(-1\right)}^{i} \end{array}$$

for *i, j* ∈ [0, *d*−1] (more details in Voelker and Eliasmith, 2018; Voelker, 2019; Voelker et al., 2019). Note that here *d* is the dimension/order of the LTI's state-space vector **x**(*t*) (in Equation 1), i.e., **x**(*t*) ∈ ℝ^*d*^ (hereby, we refer **x**(*t*) as the LDN's state-space vector). Also note that this **x**(*t*) can be used to obtain a delayed input with any delay of ϕ ∈ (0, θ) time-seconds.

#### 2.1.1. Approximating the canonical LTI system through a neural LTI system

**Figure 1:**
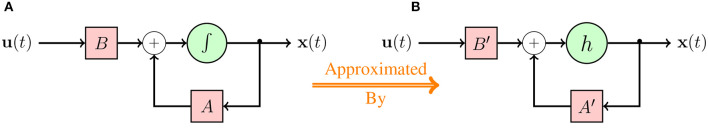
Appromixation of Equation (1a) of the Canonical LTI system through a Neural LTI system. **(A)** Canonical LTI system. **(B)** Neural LTI system.

To implement the LDN via spiking neurons in Nengo (for the SLRC model) we need to approximate the state-space (Equation 1a and Figure 1A) of the canonical LTI system in the neural domain. We can do that by approximating the integral function (Figure 1A) with a continuous-time first order low-pass filter $h\left(t\right)=\frac{1}{\tau }e^{\frac{-t}{\tau }}$ (in Figure 1B) in the neural domain; Figure 1B shows the neural LTI system described by the following equation:


(7) $$\begin{array}{l} \text{x}\left(t\right)=h\left(t\right)*\left(A^{\prime }\text{x}\left(t\right)+B^{\prime }\text{u}\left(t\right)\right) \end{array}$$

The goal is find the matrices *A*′ and *B*′, such that both the systems—the *canonical* and the *neural*, are equivalent. We can do that taking the Laplace transform of the Equations (1a) and (7), and equating them. Note that the Laplace transform of Equation (1a) is


(8) $$\begin{array}{l} s\text{X}\left(s\right)=A\text{X}\left(s\right)+B\text{U}\left(s\right) \end{array}$$

and of Equation (7) is


(9) $$\begin{array}{l} \text{X}\left(s\right)=\text{H}\left(s\right)\left(A^{\prime }\text{X}\left(s\right)+B^{\prime }\text{U}\left(s\right)\right) \end{array}$$

where $\text{H}\left(s\right)=L\left[h\left(t\right)\right]=\frac{1}{1+s\tau }$. Upon rearranging the Equation (9) (with **H**(*s*) replaced) to resemble the Equation (8), we get the following equation (proof in Supplementary material):


(10) $$\begin{array}{l} s\text{X}\left(s\right)=\frac{1}{\tau }\left(A^{\prime }-I\right)\text{X}\left(s\right)+\frac{1}{\tau }B^{\prime }\text{U}\left(s\right) \end{array}$$

Equating the coefficients of **X**(*s*) and **U**(*s*) on the RHS in both the equations (i.e., Equations 10 and 8) gives us the following values for the *A*′ and *B*′ matrices (to implement the neural LTI system):


(11a) $$A^{\prime }=\tau A+I\;\;\text{and}$$

(11b) $$B^{\prime }=\tau B$$

where *A* and *B* are defined in the Equations (6a) and (6b), respectively, and *I* is the Identity matrix.

Note that Nengo implements the discretization of continuous-time systems internally with default Δ*t* = 0.001 and ZOH method. For the LSNN model, where the LDN is implemented via regular matrix operations (in PyTorch), and not through a neural LTI system, we obtain the value of *A*′ and *B*′ matrices by explicit discretization of the continuous-time LTI system (Equation 1) with Δ*t* = 0.001 and ZOH method. Also, in both models, we refer the state-space vector **x**(*t*) as the extracted out temporal features of the input **u**(*t*).

#### 2.1.2. Tunable parameters of LDN

In the SLRC model, the tunable parameters of the LDN are its state-space vector's (i.e., **x**(*t*)'s) dimension *d* ∈ ℤ^+^, low-pass filter's (i.e., *h*(*t*)'s) time-constant τ ∈ ℝ^+^, and the length of the *rolling window* i.e., θ ∈ ℝ^+^ of the input signal **u**(*t*) which the LDN encodes in its memory. In the LSNN model, the tunable parameters of the LDN are *d* and θ only, since we do not employ neural approximation in LSNN.

### 2.2. Surrogate gradient descent (SurrGD)

As mentioned in the Section 1, direct training of SNNs with standard back-propagation algorithm is not feasible. We can use the Back-propagation Through Time algorithm to account for the temporality in SNNs, however, we also need to address the non-differentiabilty of the spikes for a successful direct training. The spiking function of a neuron *i* in layer *l*, i.e., $S_{i}^{l}\left[t\right]$ in an SNN (conventionally) depends on its membrane potential $V_{i}^{l}\left[t\right]$ and its chosen membrane threshold *V*_*thr*_; $S_{i}^{l}\left[t\right]$ can formally be written as follows:


(12) $$\begin{array}{l} S_{i}^{l}\left[t\right]=\text{Θ}\left(V_{i}^{l}\left[t\right]-V_{thr}\right) \end{array}$$

where Θ(.) is simply the Heaviside step function.

The derivative of the loss function L (of an SNN) w.r.t. weights *W* (following the chain rule) is below:


(13) $$\begin{array}{l} \frac{\partial L}{\partial W}=\underset{t}{\sum }\frac{\partial L}{\partial S\left[t\right]}\frac{\partial S\left[t\right]}{\partial V\left[t\right]}\frac{\partial V\left[t\right]}{\partial I\left[t\right]}\frac{\partial I\left[t\right]}{\partial W} \end{array}$$

One can observe in Equation (13) that the partial derivative $\frac{\partial S\left[t\right]}{\partial V\left[t\right]}$ (in light of Equation 12) is always 0, except when the argument to the Heaviside step function Θ(.) is 0, at which, the derivative is undefined; in fact, this ill defined gradient can be formulated as the Dirac's δ(*t*) function. Consequently, either the error gradients vanish in the deeper layers (when the *S*[*t*]'s derivative is 0) or explode (when the *S*[*t*]'s derivative is undefined), resulting in frozen or infinite weights, respectively.

To alleviate this problem, authors in Zenke and Ganguli (2018) and Neftci et al. (2019) discussed the usage of surrogate derivatives in place of the actual undefined derivative of *S*[*t*]. Zenke and Ganguli (2018) use the partial derivative of the negative half of the fast sigmoid function, i.e., $f^{\prime }\left(x\right)=\frac{1}{{\left(1+|x|\right)}^{2}}$ (where $x=V_{j}^{l}\left[t\right]-V_{thr}$ in our case) as a surrogate derivative; we use the same in this work. Note that this method of using surrogate derivatives to implement the gradient descent to update weights is also known as the Surrogate Gradient Descent (SurrGD) method.

Now that we have briefly explained the LDN and SurrGD, we next explain our proposed spiking models.

### 2.3. Spiking Legendre Reservoir Computing (SLRC) model

**Figure 2:**
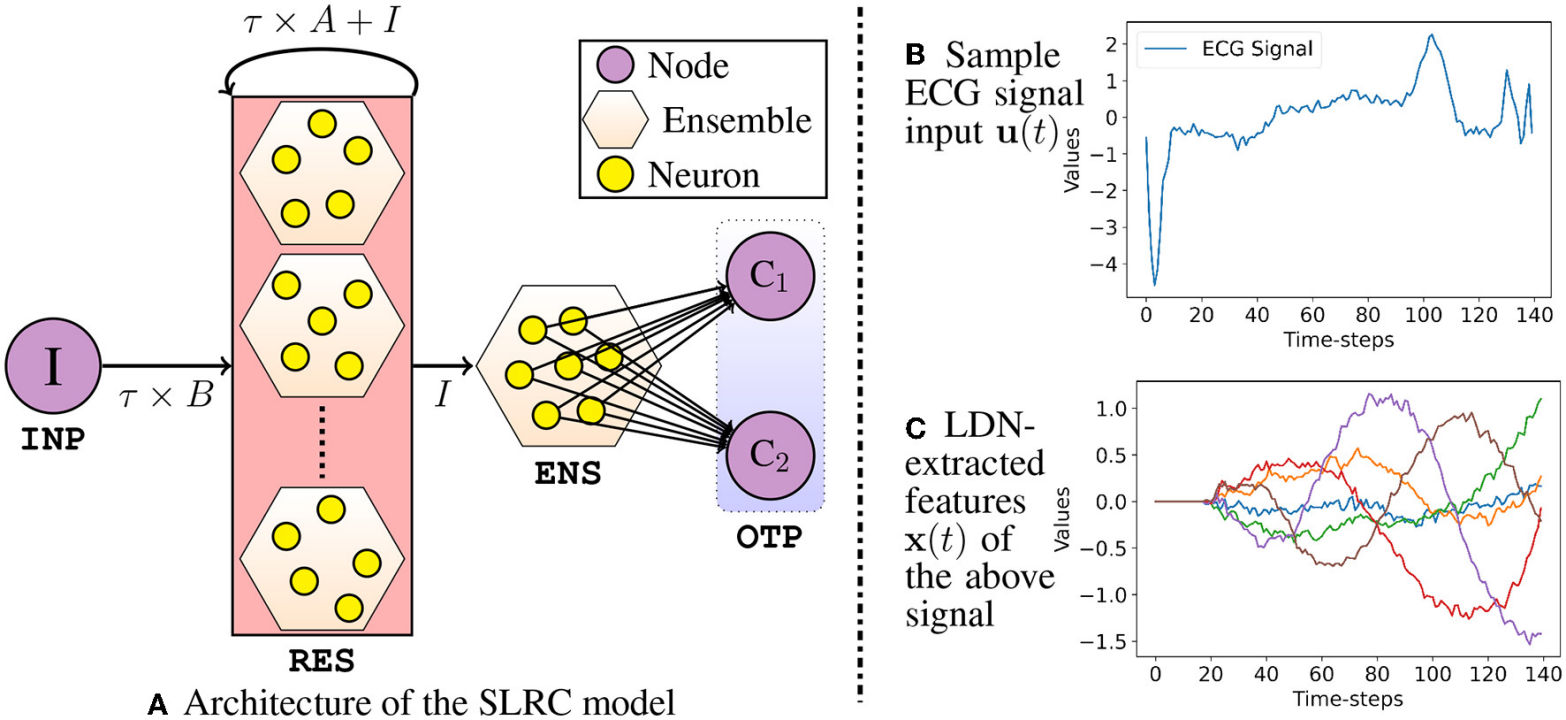
In **(A)**, **I** is the input node; **C**_**1**_, **C**_**2**_ are the output nodes. Weights between **ENS** and **OTP** are learned through regression method. Nodes are deployed off-chip; Ensembles (**RES** and **ENS**) are deployed on-chip. In **(B)**, a sample input signal **u**(*t*) is shown, and **(C)** shows the extracted **x**(*t*) from spiking LDN.

In this section, we propose and describe the architectural details of our first spiking-TSC model—the Spiking Legendre Reservoir Computing (SLRC) model. As the name suggests, this model's architecture (Figure 2A) is inspired from the conventional RC architectures, where it has an input layer, followed by a reservoir of spiking neuron ensembles, and an output layer; albeit, we add an extra ensemble of spiking neurons between the reservoir and the output layer. Note that the spiking neurons in all the ensembles in the SLRC model rate-encode the input signals. The input layer/node **INP** is connected to the reservoir **RES** with a connection weight of τ*B* (Equation 11b), and the reservoir **RES** is recurrently connected with a connection weight of τ*A*+*I* (Equation 11a). The number of ensembles in the reservoir **RES** is equal to the order of the LDN, i.e., *d*, such that each is sensitive to only one of the dimensions of **x**(*t*). Since the reservoir **RES** is further connected to an ensemble **ENS** with an identity connection weight *I*, the ensemble **ENS** simply collects and represents the extracted out *d*-dimensional temporal features, i.e., **x**(*t*), and aids in the learning of the connections to the output layer **OTP**. Note that a full connection matrix (to connect the spiking neurons) is internally obtained in Nengo by pre and post multiplying the matrices {τ*B*, τ*A*+*I*, and *I*} with a randomly generated *Encoder* matrix and a computed *Decoder* matrix, respectively. More details on the *Encoder* and *Decoder* matrices can be found in NEF (Eliasmith and Anderson, 2003; Stewart, 2012). All the connections here are static, except the connections between the **ENS** and **OTP**, which are learned offline through the Least Squares Regression method with L2 regularization.

#### 2.3.1. Network design hyper-parameters

Apart from the LDN parameters *d*, θ, and τ, other architectural parameters in the SLRC model are the number of spiking neurons, i.e., *N*_sn_ in each ensemble, their minimum and maximum firing rates, i.e., *FR*_min_ and *FR*_max_, respectively, and their representational radius *r*; more details about these parameters can be found in Eliasmith and Anderson (2003). To briefly demonstrate the LDN's state-space output **x**(*t*) for a sample input signal (Figure 2B), Figure 2C shows the extracted temporal features, i.e., **x**(*t*) for arbitrarily chosen values of *d*, τ, θ, *N*_sn_, *FR*_min_, *FR*_max_, and *r* of the **RES** ensembles. Note that these are the signals which are fed to and represented by the **ENS**, and on which the weights are learned upon by the readout layer connections to the **OTP** nodes. Note that the reservoir **RES** has *d* ensembles; and each ensemble has the same number of spiking neurons—*N*_sn_. However, since the connected ensemble **ENS** collectively represents the *d*-dimensional temporal features **x**(*t*), we set it to have a higher number of spiking neurons—$\frac{N_{sn}\times d}{4}$ (arbitrarily set, *d* > 4 while tuning). Another parameter which varies between the **RES** and the **ENS** is the radius *r*—we set it separately as *r*_RES_ and *r*_ENS_. This is done because the **RES** computes the temporal features **x**(*t*), while the **ENS** simply collects and represents those features. Note that for the time-series datasets where each sample is independent of the other and fed in online fashion to the SLRC model, one can choose to either inhibit the **RES** neurons or not inhibit it between the samples; more details in the Section 3.2. In case one chooses to inhibit the **RES**, two architectural parameters need to be set—the *magnitude* and the *duration* of the inhibition.

### 2.4. Legendre Spiking Neural Network (LSNN) model

We next propose and describe the architectural details of our second spiking-TSC model—the Legendre Spiking Neural Network (LSNN) model. Similar to the SLRC model, this model uses the LDN to extract the high dimensional temporal features **x**(*t*) from the input signal **u**(*t*). Before diving into the architecture of the LSNN model, let us define the neuron's membrane potential (*V*(*t*)) and current (*I*(*t*)) state equations (in continuous-time domain), used in our LSNN model.

#### 2.4.1. IF neuron's state equations

Equations (14a), (14b), and (15) define the IF neuron state equations (*i* is the index of the neuron in layer *l*):


(14a) $$\begin{array}{ll} \frac{dV_{i}^{l}(t)}{dt} & =\frac{RI_{i}^{l}(t)}{\tau_{\text{vol}}}\;\;\;\;\;\;\text{when }V_{i}^{l}(t)<V_{thr} \end{array}$$

(14b) $$\begin{array}{ll} V_{i}^{l}(t) & \leftarrow 0\;\;\;\;\;\;\;\;\text{when }V_{i}^{l}(t)\geq V_{thr} \end{array}$$

     and


(15) $$\begin{array}{l} \frac{dI_{i}^{l}(t)}{dt}=\frac{-I_{i}^{l}(t)}{\tau_{\text{cur}}}+\sum_{j}W_{j,i}^{l}S_{j}^{l-1}(t) \end{array}$$

where τ_vol_ and τ_cur_ are the membrane voltage and current time constants, respectively, and *R* is the membrane resistance. Our state equations differ from Neftci et al. (2019) in ways that we do not consider the leak term (Burkitt, 2006) in the ODE for *V*(*t*) (Equation 1 in Neftci et al., 2019) and the recurrent term in the ODE for *I*(*t*) (Equation 2 in Neftci et al., 2019). Also, we conveniently set the value of *R* to 1 and keep the time constants tunable. We define the discrete-time equations later.

#### 2.4.2. LSNN architecture

**Figure 3:**
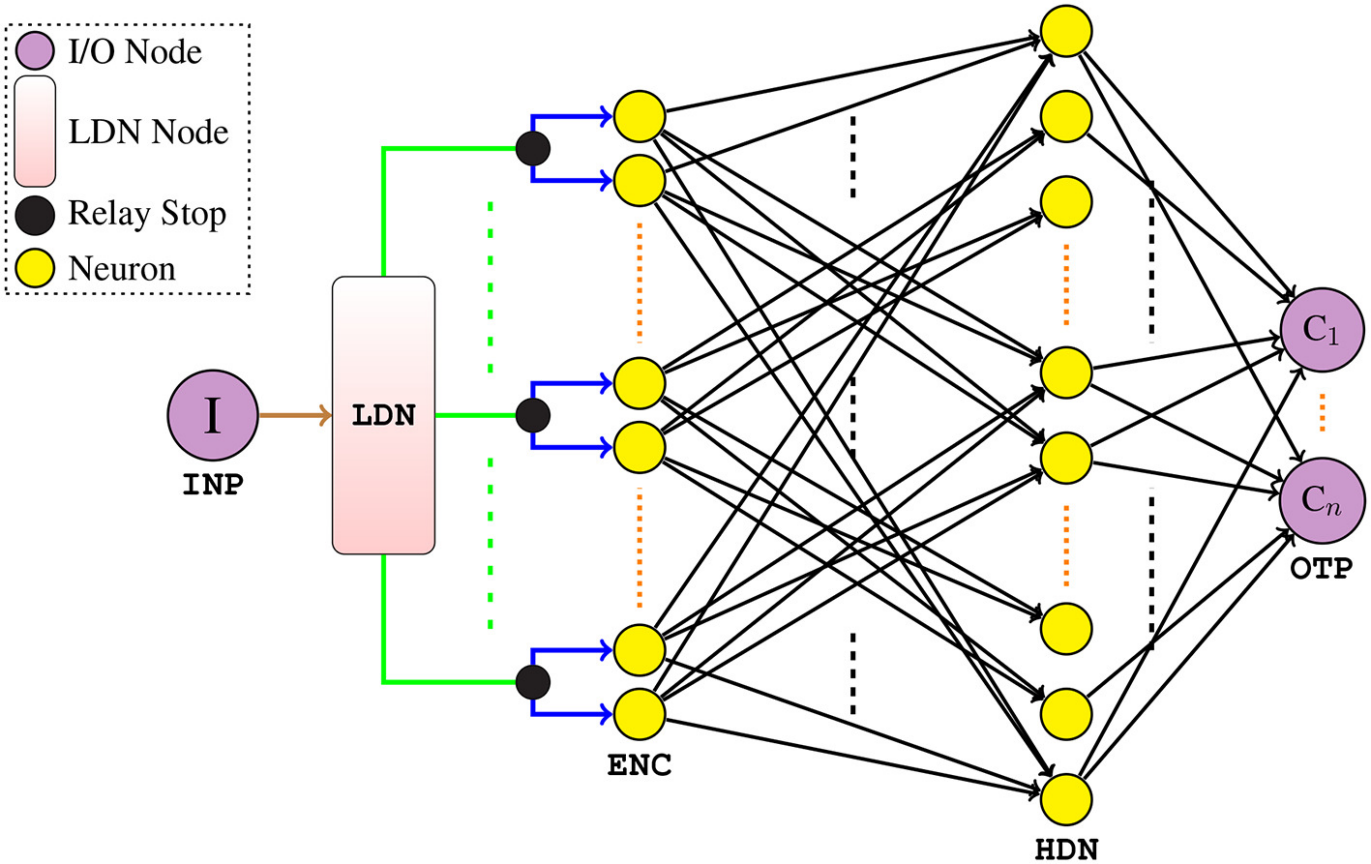
Architecture of our LSNN model (all neurons are IF spiking). The **INP** is connected to the **LDN** through a static connection weight of 1. The outputs from the **LDN**, i.e., **x**(*t*) (the green connections) are relayed as is to the relay stops where each individual feature signal is multiplied by the static weights of 1 and 1 (simultaneously), i.e., the blue connections and fed to **ENC**. Only the connections in black are trained.

Now that we have formally described the neuron state equations, we next describe the architecture of our proposed LSNN model—Figure 3. It comprises of an input node **I** (**INP**) to feed the input signal **u**(*t*) to the network, followed by the **LDN** node—which constitutes of static linear matrix operations. The **LDN** maps the univariate input to the *d*-dimensional temporal features **x**(*t*), which are then relayed to the IF neurons (in pairs) in the **ENC** layer; as the name suggests, the **ENC** layer neurons rate-encode the extracted **x**(*t*) to binary spikes. Every pair of the IF neurons in the **ENC** layer consists of a positive encoder and a negative encoder—which encodes the positive and negative part of the feature signal **x**(*t*), respectively. Note that only one of the encoder neurons in each pair is active at a time. The **ENC** layer neurons are next densely connected to the **HDN** layer of IF neurons through an all-to-all connection. Note that there is only one **HDN** layer of IF neurons which is then densely connected to the **OTP** layer (where **C**_***i***_ are class nodes). In our model, only the connections between the **ENC** layer to **HDN** layer and the **HDN** layer to **OTP** layer are trained; rest of the connections remain static. Also, the usage of surrogate derivatives in the **HDN** layer enables the error gradient flow backwards to the **ENC** layer, thereby updating the hidden layer connections.

#### 2.4.3. Discrete-time state equations

Here, we describe the discrete-time functioning of the LSNN components. For simulation ease, we first extract and save the temporal features of the entire training and test set, from the LDN. We then simulate the rest of the network, i.e., the spiking part with extracted temporal features as the input (green connections) to the **ENC** layer. Note that in each simulation time-step, the entirety of the spiking network is simulated, which enables us to have current time-step values from the previous layers (more details in sections below).

##### 2.4.3.1. **ENC** layer

Encoding neurons in the **ENC** layer follow the state equations defined below (here *l* is **ENC** layer only):


(16a) $$I_{i}^{l}[t]=\rho \times \epsilon_{i\;\mathrm{mod}\;2}\times x_{\left\lfloor \frac{i}{2}\right\rfloor }[t]+\iota$$

(16b) $$V_{i}^{l}[t]=V_{i}^{l}[t-1]+I_{i}^{l}[t]$$

where *i* ∈ [0, 1, ⋯ , 2*d*−1], ρ and ϵ_*i* mod 2_ are the IF neuron's gain and encoder values, respectively, where ϵ_0_ = 1 and ϵ_1_ = −1, and ι is the bias current (Eliasmith and Anderson, 2003). **x**[*t*] ∈ ℝ^*d*^ is the temporal feature vector from the LDN. Note that when *V*[*t*] reaches the threshold *V*_thr_, a spike is generated (Equation 12) and it is reset to 0 (Equation 14b).

##### 2.4.3.2. **HDN** layer

**HDN** layer IF neurons follow the state equations defined below (*l* is one **HDN** layer in LSNN model) :


(17a) $$I_{i}^{l}[t]=\alpha I_{i}^{l}[t-1]+\sum_{j}W_{j,i}^{l}S_{j}^{l-1}[t]$$

(17b) $$\begin{array}{ll} V_{i}^{l}\left[t\right] & =V_{i}^{l}\left[t-1\right]+I_{i}^{l}\left[t\right] \end{array}$$

where α is a current decay constant ($\alpha =\text{exp}\left(\frac{-\text{Δ}t}{\tau_{\text{cur}}}\right),\text{Δ}t=0.001$). Here, too $V_{i}^{l}\left[t\right]$ is reset once it reaches the threshold *V*_thr_ (Equation 14b) and a binary spike is generated (Equation 12).

##### 2.4.3.3. **OTP** layer

The **OTP** layer nodes function similar to the IF neurons in the **HDN** layer, except that they do not output a spike and their voltage decays with time (hence, we do not qualify them as IF neurons); they follow the equations below (here *l* is just the **OTP** layer):


(18a) $$I_{i}^{l}[t]=\alpha I_{i}^{l}[t-1]+\sum_{j}W_{j,i}^{l}S_{j}^{l-1}[t]$$

(18b) $$V_{i}^{l}[t]=\beta V_{i}^{l}[t-1]+I_{i}^{l}[t]$$

where β is a voltage decay constant ($\beta =\text{exp}(\frac{-\text{Δ}t}{\tau_{\text{vol}}}),\text{Δ}t=0.001$). Note that Equation (18a) and (17a) are same. We define the classification loss function on the maximum voltage of the output nodes (over all the simulation time-steps). We use the PyTorch's negative log likelihood loss function, i.e., NLLLoss(ŷ_pred_, *y*_true_) to calculate the loss, where *y*_true_ are the true classes and ŷ_pred_ = log(*softmax*(*x*)), $x=\underset{t}{\max}(V^{l}[t])$.

#### 2.4.4. Network design hyper-parameters

The number of **ENC** layer neurons depends on the order *d* of the LDN, i.e., *N*_ENC_ = 2 × *d*. We arbitrarily set the number of **HDN** layer neurons to *N*_HDN_ = 3 × *d*. The number of **OTP** layer nodes is equal to the number of classes, which is 2 in all our experimented datasets. With respect to the LDN, as mentioned in Section 2.1.2, only *d* and θ are tunable. With respect to the neurons in the **ENC** layer, their *gain* and *bias* values, i.e., ρ and ι, respectively, are kept tunable. The neurons in the **HDN** layer have their τ_cur_ set tunable. In addition, all the IF neurons (in the **ENC** and **HDN** layers) have their voltage decay constant β = 1 (i.e., no voltage decay) and *V*_thr_ set tunable. In the **OTP** layer, the voltage time-constant τ_vol_ is kept tunable, thus the voltage decay constant β is tunable. Overall, the following hyper-parameters were tuned during our LSNN experiments: LDN dimension *d*, rolling window θ (in seconds), gain ρ, bias ι, τ_cur_, τ_vol_, and *V*_thr_.

## 3. Experiments

In this section, we outline the implementation level details of our proposed models and the conducted experiments, and finally present the accuracy results. We also detail out the derivative models of the LSNN model with which compare/benchmark against. We start with the datasets description, followed by the training, deployment, and evaluation details of the SLRC and the LSNN model, followed by the results.

### 3.1. Datasets

We train and evaluate our models on univariate binary-TSC datasets. For the SLRC model, we use only the ECG5000 dataset; and for the LSNN model, we use the ECG5000 along with four others, experimented with in Dey et al. (2022)—Ford-A, Ford-B, Wafer, and Earthquakes. We chose these datasets not only to compare our results with, but also to show the application of our models in sensor domain. We briefly describe each of the five datasets next. Note that all these datasets are available at the Time Series Classification website.^1^

#### 3.1.1. ECG5000

This dataset consists of 500 training samples and 4, 500 test samples of ECG signals; each sample $s_{i}\in {\mathbb{R}}^{140}$. The dataset has 5 classes: *N*, *R-on-T* *PVC*, *PVC*, *SP*, and *UB*—class definitions are in Table 1 that also shows the sample distribution and sample counts for all the 5 classes. As can be inferred from the Table 1, the ECG5000 dataset is heavily imbalanced. Note that the class *N* corresponds to a normal/healthy heartbeat, and rest of the classes correspond to abnormal/unhealthy heartbeat. We therefore group all the 4 abnormal classes into one class. Thus, the ECG5000 time-series classification task is modeled as a binary classification task between healthy and unhealthy heartbeats; a few authors do the same (Matias et al., 2021; Oluwasanmi et al., 2021; Biloborodova et al., 2022).

**Table 1:** Training and test samples distribution and count of the ECG5000 dataset.

**Class**	**Training set**	**Test set**
Normal (*N*)	58.4% (292)	58.38% (2627)
R-on-T premature ventricular contraction (*R-on-T PVC*)	35.4% (177)	35.33% (1590)
Premature ventricular contraction (*PVC*)	2.00% (10)	1.91% (86)
Supra-ventricular premature beat (*SP*)	3.80% (19)	3.89% (175)
Unclassified beat (*UB*)	0.40% (2)	0.49% (22)

#### 3.1.2. Ford-A

This dataset consists of 3, 601 training and 1, 320 test samples of the engine noise signals; each sample $s_{i}\in {\mathbb{R}}^{500}$. The task is to diagnose whether or not a certain disorder exists based on the engine noise—thus, a binary TSC problem. We discard the last 1 training sample to suit the experiment's batch requirements.

#### 3.1.3. Ford-B

It is similar to Ford-A, except that while the training data of 3, 636 samples were collected under typical operating conditions, the test data of 810 samples were collected under noisy conditions. Note that here too, each sample $s_{i}\in {\mathbb{R}}^{500}$, and the task is to identify whether or not a disorder exists in the engine subsystem.

#### 3.1.4. Wafer

This dataset consists of 1, 000 training samples and 6, 164 test samples of the sensor signal recorded during the processing of wafers; each sample $s_{i}\in {\mathbb{R}}^{152}$ and belong to either normal or abnormal class, thus, a binary TSC task. We discard the last 14 test samples to suit the experiment's batch size requirements.

#### 3.1.5. Earthquakes

This dataset consists of 322 training and 139 test samples of the recorded seismic data; each sample $s_{i}\in {\mathbb{R}}^{512}$. The task is to predict whether or not a major event is about to occur based on the seismic data—thus, a binary TSC task. We discard the last 1 test sample to suit the experiment's batch size requirements.

### 3.2. SLRC model training and evaluation

We next describe the experiment details of our proposed SLRC model. We start with the hyper-parameter tuning details, followed by the deployment details on the Loihi neuromorphic hardware and the CPU.

#### 3.2.1. Hyper-parameter tuning

As noted earlier, for the SLRC model, we experimented with just the ECG5000 dataset, where each sample is independent of the other. For such disjointed ECG signals each having their own class, through some preliminary experiments we found that inhibition of the **RES** neurons between the samples while training helps in achieving higher training and test accuracy. This is because the inhibition of the **RES** neurons helps it clear out the memory of the previous input signal. Therefore, following the basic investigative experiments, we set the *magnitude* and the *duration* of the inhibition to be 8 and 50 time-steps, respectively. In addition to setting the inhibition parameters, we also set the τ parameter to 0.1—we found that varying it doesn't improve the results significantly. Next, we begin training our SLRC model along with tuning the rest of the hyper-parameters. Table 2 shows the hyper-parameter values over which the grid-search is done—for both the platforms: Loihi and CPU. For each of the platforms, we choose the hyper-parameter combination for inference which provides the best training accuracy. Note that, to account for the random initialization of the Nengo networks, we conduct grid-search with two different SEED values (3 & 9). Also note in Table 2, that θ is limited to maximum 0.14s, since each ECG signal is only 140 time-steps long and we consider 1 time-step = 1ms. We next present the platform specific deployment details.

**Table 2:** Hyper-parameter values over which grid-search is done for the SLRC model on ECG5000 dataset.

**Hyper- parameters**	**Deployment platforms**	
	**Values on Loihi**	**Values on CPU**
*d*	{6, 8, 10}	{6, 8, 10}
θ	{0.10, 0.12, 0.14}	{0.12, 0.14}
*FR*_*min*_	{40, 60, 80}	{75, 150}
*FR*_*max*_	{100, 120, 140}	{250, 350}
*r*_RES_	{0.5, 1.0, 1.5}	{0.5, 1.0, 1.5, 2.0}
*r*_ENS_	{0.5, 1.0, 1.5}	{0.5, 1.0, 1.5, 2.0}
*N*_*sn*_	{100, 200}	{100, 200}

#### 3.2.2. Loihi-1 deployment

We use the NengoLoihi simulator to deploy our SLRC model on the Loihi-1 boards. Note that on Loihi-1, the IF neurons suffer from firing rate quantization errors, i.e., their firing rates are quantized. This is because the spikes on Loihi-1 are binary, i.e., they assume a value of either a 0 or a 1. This coupled with IF neurons having integral Inter Spike Interval (ISI), results in them to fire only at certain designated firing rates, e.g., at 500 Hz (when ISI = 2), 333 Hz (when ISI = 3), 250 Hz (when ISI = 4), 200 Hz (when ISI = 5), and so on... This unfortunately limits their ability to differentiate between multiple inputs (more details later). Consequently, the spiking networks on Loihi-1 have poor expressivity and limited discriminatory power. Therefore, to limit the effects of quantization errors, we train our SLRC model with low minimum and maximum firing rates for Loihi deployment. The accuracy results are mentioned in the Table 3.

**Table 3:** ECG5000 test accuracy with corresponding hyper-parameters.

**SLRC Model Parameters**								**Test accuracy - on platform**
**SEED**	*N*_sn_	*FR*_min_	*FR*_max_	*d*	θ	*r*_RES_	*r*_ENS_	
9	100	80	120	6	0.14	1.5	0.5	80.20% - on Loihi
3	200	150	350	10	0.12	0.5	1.0	91.97% - on CPU

#### 3.2.3. CPU deployment

We use the Nengo simulator to deploy our SLRC model on CPUs. Note that on CPUs, the IF neurons do not suffer from firing rate quantization errors. This is because the spikes on CPUs can be graded, which allows the IF neurons to have a continuous spectrum of firing rates (more details later). Consequently, they can distinctively represent each input, and the IF neurons based spiking networks on CPUs have better expressivity and higher discriminatory power. Therefore, we perform grid-search with higher minimum and maximum firing rates on CPUs than on Loihi. The accuracy results are mentioned in Table 3.

### 3.3. LSNN model training and evaluation

Here, we describe the experiment details of our proposed LSNN model. We start with its hyper-parameter tuning details, followed by that of its derivative models. We then present the results obtained with LSNN and its derivatives on the five TSC datasets. Note that we do not deploy this model on Loihi, it was trained and evaluated on CPUs only. We used the Adam optimizer (Kingma and Ba, 2014) for all our LSNN related experiments.

#### 3.3.1. Hyper-parameter tuning

As mentioned in Section 2.4.4, we fix the number of neurons in the **ENC** and **HDN** layers to 2 × *d* and 3 × *d*, respectively, and the tunable parameters in the LSNN model are *d*, θ, ρ, ι, τ_cur_, τ_vol_, and *V*_thr_—which we tune differently in cognizance of the datasets; in addition, we also tune the learning rate η. The number of training epochs and batch size too, varies with the dataset; they are, *training epochs*: 50 for ECG5000 and Wafer, 250 for Ford-A, Ford-B, and Earthquakes, and *batch size*: 50 for ECG5000 and Wafer, 40, 18, 23 for Ford-A, Ford-B, and Earthquakes, respectively. For a dataset, the number of epochs and batch size are kept the same across all its related experiments. Note that, unlike the case with SLRC, there is no need to inhibit the LDN here, since it is implemented via the matrix operations (in the LSNN model) and there is no residual memory of the previous input. We conducted a few preliminary LSNN experiments on the ECG5000 dataset to investigate the effects of the tunable hyper-parameters; which we later adapt to all the datasets and conduct our exhaustive experiments. During the preliminary experiments with ECG5000, we also found that the hard reset of *V*[*t*] and no normalization of the LDN extracted features **x**(*t*) improve the inference accuracies; therefore, we keep this setting for the rest of the datasets. Table 4 shows the dataset specific hyper-parameters' values over which the grid search is done—for three different runs with SEED ∈ {6, 9, 100}. Note that we shuffle the training data every 20 epochs for all the experiments, and calculate the test accuracy on the entire test set every training epoch. Table 5 shows the test accuracy results for all the experimented datasets, obtained over all the (dataset specific) hyper-parameter combinations and the three SEED values; we provide further explanations of Table 5 in Section 4.

**Table 4:** Hyper-parameters values over which the grid-search is done for the LSNN experiments, for each of the three runs with different SEED values.

**Hyper- params**	**Datasets**				
	**ECG5000**	**WAFER**	**FORD-A**	**FORD-B**	**EARTHQUAKES**
*d*	{12, 14}	{10, 12, 14}	{10, 12, 16, 24}	{10, 12, 16, 24}	{10, 12, 16, 24}
θ	{0.12}	{0.11, 0.13, 0.15}	{0.025, 0.05, 0.1, 0.15}	{0.025, 0.05, 0.1, 0.15}	{0.025, 0.05, 0.1, 0.15}
ρ	{1, 2, 4}	{1, 2, 4}	{2, 4}	{2, 4}	{2, 4}
ι	{0, 0.5}	{0, 0.5}	{0, 0.5}	{0, 0.5}	{0, 0.5}
τ_cur_	{5e-3, 10e-3, 15e-3}	{5e-3, 10e-3, 15e-3}	{5e-3, 10e-3}	{5e-3, 10e-3}	{5e-3, 10e-3}
τ_vol_	{10e-3, 20e-3, 30e-3}	{10e-3, 20e-3, 30e-3}	{20e-3, 30e-3}	{20e-3, 30e-3}	{20e-3, 30e-3}
*V*_thr_	{1, 1.5}	{1, 1.5}	{1, 1.5}	{1, 1.5}	{1, 1.5}
η	{0.005, 0.01, 0.05}	{0.005, 0.01, 0.05}	{0.005, 0.01}	{0.005, 0.01}	{0.001, 0.005, 0.01}

In case of Ford-A, Ford-B, and Earthquakes, the combinations of *d* = {10, 12} with θ = {0.025, 0.05, 0.1}, and *d* = {16, 24} with θ = {0.1, 0.15} were considered.

**Table 5:** Comparison of test accuracy results (in %) obtained from our LSNN model and its derivatives.

**Dataset**	**Non-spiking Acc. (SoTA)**	**Spiking Acc. (SoTA)**	**LSNN Acc**.		**LSNN**_**nhdn**_ **Acc**.		**LSNN**_**nspk**_ **Acc**.	
			**Max**_acc_	**Mean**_acc_	**Max**_acc_	**Mean**_acc_	**Max**_acc_	**Mean**_acc_
**ECG5000**	*98.43* (Pereira and Silveira, 2019)	–	**98.49**	98.19 ± 0.21	97.73	97.22 ± 0.37	*98.42*	*98.18 ± 0.18*
**WAFER**	*100.00* (Karim et al., 2017)	98.85 (Dey et al., 2022)	**99.51**	99.38 ± 0.17	99.38	99.30 ± 0.06	*99.82*	*99.76 ± 0.05*
**FORDA**	*97.33* (Karim et al., 2017)	80.37 (Dey et al., 2022)	**93.56**	93.36 ± 0.19	89.62	88.25 ± 1.67	*93.03*	*92.98 ± 0.07*
**FORDB**	*92.86* (Lines et al., 2018)	64.32 (Dey et al., 2022)	**82.72**	81.98 ± 0.88	77.78	76.46 ± 1.06	*81.98*	*81.90 ± 0.12*
**EARTHQUAKES**	*83.54* (Karim et al., 2017)	71.94 (Dey et al., 2022)	**80.43**	79.95 ± 0.68	79.71	78.74 ± 1.37	*81.88*	*79.71 ± 1.56*

**Acc**. stands for accuracy; **Max**_**acc**_ and **Mean**_**acc**_ stand for the maximum accuracy and mean of the maximum accuracies (mean ± std) over 3 runs. We compare our results with only the SoTA spiking ones (Dey et al., 2022). The italicized values indicate accuracy values from non-spiking models. The bold values indicate the highest accuracy results.

#### 3.3.2. Derivative models of LSNN

In this section, we do the ablation study of the LSNN model. We next explain the derivative models of the LSNN which were employed to characterize the practicality of the LSNN's architecture. The first derivative of LSNN is obtained by removing the spiking hidden layer (**HDN**) altogether—to resemble the conventional RC architecture, and the second one is obtained by switching the spiking neurons to the non-spiking ReLU neurons. Note that for all the derivatives' experiments with each dataset, we use the related hyper-parameters' values mentioned in the Table 4, and execute three runs with SEED ∈ {6, 9, 100}.

##### 3.3.2.1. No hidden layer

To qualify if the **HDN** layer of spiking neurons is necessary in the LSNN model's architecture for a superior performance, we compare our LSNN against its derivative model with no **HDN** layer; such that the derivative now resembles a conventional RC architecture. It now has the **LDN** which maps the input to the high dimensional temporal features **x**(*t*), followed by the **ENC** layer of 2 × *d* encoding neurons densely connected to the **OTP** layer, i.e., with no non-linearity in between. We call this model as LSNN_nhdn_.

##### 3.3.2.2. Non-spiking model

To qualify how the LSNN performs against its non-spiking counterpart, we replace the spiking neurons in the LSNN's architecture with non-spiking ReLU neurons. Since the ANNs do not need to separately encode the positive and negative part of the input, we replace the **ENC** layer with an Input layer of *d* nodes, followed by a Hidden layer of 3 × *d* number of ReLU neurons. In the Output layer, we collect the outputs from the class nodes over all the simulation time-steps and apply the same procedure as in Section 2.4.3.3 to calculate the loss and back-propagate it. We call this non-spiking variant as LSNN_nspk_.

Table 5 shows the results obtained with the LSNN_nhdn_ and LSNN_nspk_ models, in comparison to the other SoTA results. Code at: https://github.com/R-Gaurav/spiking-models-for-TSC.

## 4. Result analysis

Here, we present a detailed analysis of the experiment results obtained from our models: the SLRC and the LSNN models. We start with SLRC model's result analysis followed by that of the LSNN.

### 4.1. SLRC model's result analysis

The test accuracy results mentioned in Table 3 are obtained from the hyper-parameter combination (refer Table 2) which gave the best training accuracy. As can be seen in the Table 3, we obtained 80.20% and 91.97% test accuracy on the ECG5000 dataset, on Loihi and CPU, respectively. Note that we rounded off the class prediction scores to calculate accuracy. To the best of our knowledge, our spiking results for the ECG5000 dataset with neuromorphic deployment are the first, therefore, we could not compare our results with any. We present a comparison with non-spiking results on the ECG5000 dataset later. As can be seen, the spiking results on CPU are higher than that on Loihi. This is due to the IF neurons having a more continuous spectrum of firing rates on CPU than on Loihi—explained in detail in the next paragraph.

#### 4.1.1. Quantization error comparison on CPU and Loihi

**Figure 4:**
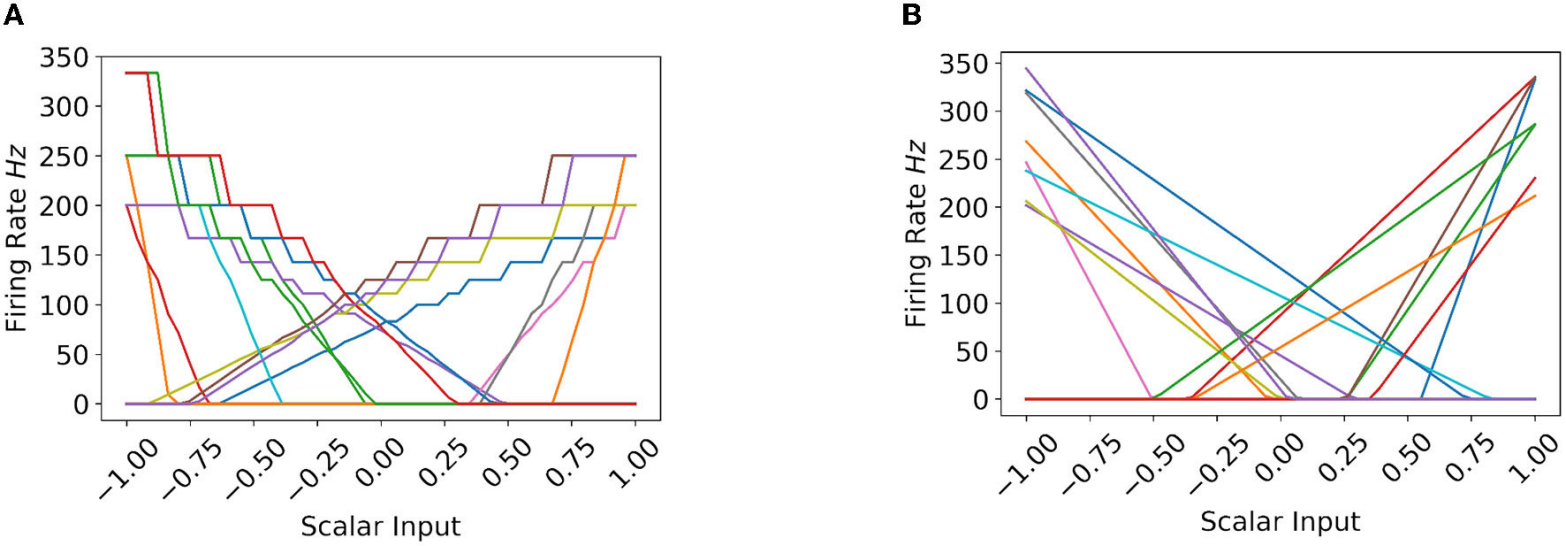
**(A, B)** Show the firing rate profile of IF neurons on Loihi and CPU, respectively. Note *r* = 1. **(A)** Tuning Curves on Loihi. **(B)** Tuning Curves on CPU.

To better illustrate the firing rate quantization error difference on Loihi-1 and CPU, Figure 4 shows the tuning curves (or the firing rate profiles) of 15 random IF neurons on Loihi-1 and CPU. As can be seen in case of Loihi-1 (Figure 4A), the tuning curves are quite smooth only until 100 Hz, beyond which they are jagged. That is, the firing rates on Loihi are quantized—multiple scalar inputs elicit the same firing rate value, and the quantization effect on firing rates becomes more prominent after 100 Hz or so. Therefore, we keep the IF neurons' firing rates on Loihi around 100 Hz. However, in case of CPU (Figure 4B), the tuning curves are smooth all along the firing rate spectrum. That is, each scalar input elicits different firing rate values. Therefore, we can leverage this higher variability to our advantage by keeping higher minimum and maximum firing rates, as well as by utilizing a broader spectrum.

With respect to improving the results on Loihi, we tried to further fine tune the hyper-parameters around the values mentioned in the Table 3 (with respect to Loihi experiments)—*FR*_*max*_ ∈ [110, 112, ⋯ , 130], *FR*_*min*_ ∈ [70, 72, ⋯ , 90], *r*_RES_ ∈ [1.1, 1.2, ⋯ , 2.0], *r*_ENS_ ∈ [0.1, 0.2, ⋯ , 0.9]—one parameter at a time in an arbitrary order. However, fine tuning on Loihi did not improve the results than already mentioned in the Table 3. One specific trend that we did observe between the accuracy results and the order *d* of the LDN (keeping the rest of the hyper-parameters same), is that on CPU, the results improved with the increase in *d*, but on Loihi, the results worsened; this worsening can be attributed to the firing rate quantization effect on Loihi. Higher *d* implies richer temporal information in the state-space vector **x**(*t*); however, due to the limited representational capacity of the spiking networks on Loihi-1, they could not leverage it and the models overfitted. This wasn't the case with the spiking networks on CPU, which very well differentiated and leveraged the high dimensional state-spaces to improve the classification accuracy, with increase in *d*.

### 4.2. LSNN model's result analysis

We start by rementioning that for each dataset, we obtained the prediction accuracy on the entire test set every training epoch, and the ECG5000 dataset was molded to suit the binary TSC task; therefore, we compare our results on it with works which do the same. The results reported in the Table 5 under the LSNN's and its derivative models' columns are defined as follows: **Max**_**acc**_ denotes the maximum test accuracy obtained over all the hyper-parameter combinations (dataset specific, refer Table 4) across all the three runs with different SEED values, and **Mean**_**acc**_ denotes the mean of the maximum test accuracies (over all the dataset specific hyper-parameter combination) obtained in each of the three runs. We also mention the State-of-The-Art (SoTA) results obtained with the non-spiking methods [e.g., LSTM-FCN (Karim et al., 2017) and HIVE-COTE (Lines et al., 2018)] for completeness, but compare ours with only the other spiking results for fairness. As can be seen in the Table 5, the **Max**_**acc**_ results obtained with the LSNN model completely outperforms the latest spiking results on the Wafer, Ford-A, Ford-B, and the Earthquakes dataset reported by Dey et al. (2022). In fact, with the LSNN model, considering the **Max**_**acc**_ results, we get an improvement of 0.668%, 16.412%, 28.607%, and 11.802% in classification accuracy (over Dey et al., 2022) for Wafer, Ford-A, Ford-B, and Earthquakes datasets, respectively. On the ECG5000 dataset, the LSNN model obtains a maximum accuracy of 98.49% which interestingly outperforms the current SoTA 98.43% obtained by a non-spiking model (Pereira and Silveira, 2019), although, by a small margin. Also note that the **Mean**_**acc**_ results under the LSNN column are very close to the **Max**_**acc**_ results (also under LSNN) with minimal standard deviation, while also being better than those obtained by Dey et al. (2022). We next analyse the accuracy results obtained by the LSNN model's derivatives.

Considering the **Max**_**acc**_ and **Mean**_**acc**_ accuracy results obtained with the LSNN_nhdn_ model (where the network is still spiking, but with no **HDN** layer), we see that they too outperform the results obtained by Dey et al. (2022), with 0.536%, 11.509%, 20.927%, and 10.801% improvement (w.r.t. **Max**_**acc**_ results under LSNN_nhdn_) on Wafer, Ford-A, Ford-B, and Earthquakes datasets, respectively. Note that the **Max**_**acc**_ and **Mean**_**acc**_ results under the LSNN_nhdn_ column are relatively poorer than those obtained with the LSNN model (especially for Ford-A and Ford-B); this implies that non-linearity in the readout layer (i.e., the **HDN** layer) is necessary for a superior performance. Next, considering the **Max**_**acc**_ and **Mean**_**acc**_ accuracy results obtained with the LSNN_nspk_ model, where the spiking neurons are replaced with non-spiking ReLU neurons, it is encouraging to note that the LSNN model performs either similar or better than its non-spiking counterpart. Generally, ANN-to-SNN conversion does not yield a superior performing SNN (than its isomorphic ANN). This seconds the strength of the SurrGD approach to train the SNNs from scratch, such that the training takes into account and leverages the temporal dynamics of the SNN. Note that our non-spiking LSNN model (i.e., LSNN_nspk_) does not outperform the SoTA non-spiking results. This can be attributed to the simplicity of our LSNN_nspk_'s architecture compared to that of the complex models such as LSTM-FCN.

Similar to the SLRC experiments on CPUs, we observed that the performance of LSNN model improves with the increase in *d* [of the LDN's state-space output **x**(*t*)]. This can be due to two reasons here: (1) richer temporal information to train and infer upon, and (2) increased number of neurons in the architecture. We also note that our LSNN's neurons generate binary spikes, thus, they too suffer from the firing rate quantization error. However, as seen in the Table 5 with respect to the ECG5000 dataset, the performance of LSNN and LSNN_nhdn_ is better than that of the SLRC model on Loihi (and even on CPU). This positive difference could be due to the exact *calculation* of the state-space vector **x**(*t*) in LSNN (and LSNN_nhdn_), in contrast to its *approximation* in the SLRC model—as the LDN in the SLRC model is implemented via spiking neurons (both on Loihi and CPU). This *approximation* coupled with the firing rate quantization error on Loihi-1, severely limits the expressivity and discriminatory power of the LDN extracted features **x**(*t*). With respect to SLRC on CPU, although the spikes are graded, the state-vector **x**(*t*) is still *approximated*, and further *represented* via the **ENS** which contributes to added information loss in **x**(*t*) (also true in case of Loihi deployment). We also surmise that iterative SurrGD in LSNN_nhdn_ perhaps offers a better fit to the data than the Least Squares Regression fitting in SLRC, although, this should be thoroughly investigated.

## 5. Discussion

We now present a detailed discussion on our proposed models, starting with the SLRC model, followed by the LSNN model. We then present the energy consumption analysis of our models on CPU and Loihi-1.

### 5.1. Discussion on the SLRC model

In light of the limitations of the SLRC model described in the paragraph above, one may question the necessity of *approximating* and further *representing* the temporal features **x**(*t*) via spiking neurons in the model. This was done because of the following reasons:

- First, the SLRC model serves the purpose to show that the LDN can be entirely implemented with spiking neurons, and a spiking RC based model can be built with it
- Second, following the NEF theory, the *approximation* and *representation* of vectors can be improved by increasing the number of neurons in the ensembles
- Third, although further *representation* of the *approximated* temporal features **x**(*t*) could have been avoided by having the **RES** constituted directly of spiking neurons (and connecting the **OTP** nodes to the **RES** neurons directly), the architectural decision to break the **RES** into ensembles and then collectively *represent* the **x**(*t*) via another **ENS** was taken due to the following two sub-reasons:
  - No separate ensembles in the **RES** would mean that all the neurons would be sensitive to all the dimensions of **x**(*t*), thereby poorly approximating the state-space vector (for the same total number of neurons as with splitting the **RES** into ensembles—each composed of lesser number of neurons)
  - The number of learnable readout connections from the **RES** neurons to the **OTP** nodes would be too high, thereby increasing the SLRC model's complexity

Therefore, where the *approximation* of the temporal features **x**(*t*) is implicit due to the spiking implementation of the LDN, further *representing* it via a smaller **ENS** of neurons lowers down the SLRC model's complexity. We note that the SLRC model, compared to the LSNN is quicker to train (due to the Least Squares Regression method) and more neuromorphic-hardware friendly (as demonstrated by us via its deployment and inference on Loihi-1). However, to overcome the limitations of the *approximation* and *representation* of **x**(*t*), thereby increasing the SLRC model's performance, one would be needed to employ ensembles with large number of neurons—but this comes at the cost of higher computational complexity.

We further note that Loihi-2 [which has been recently released (Orchard et al., 2021)] overcomes the binary spikes limitation of Loihi-1 by offering 32-bit graded spikes implementation, similar to CPU. Therefore, we expect that our SLRC model when deployed on the Loihi-2 boards, would achieve similar test accuracy results as that on CPU. Also note that in our SLRC model, the inhibition of the **RES** neurons is not always necessary. For a continuous stream of inputs, i.e., for an online input signal where the class of a current scalar input depends on the previous inputs, intervening inhibition would be adverse.

### 5.2. Discussion on the LSNN model

To mitigate the performance extenuating effect of the *approximation* and *representation* of the temporal features **x**(*t*) by the spiking LDN, we decided to explicitly calculate **x**(*t*) through regular matrix operations in the LSNN model. This helps in two ways. First, the accurate and information-rich temporal features **x**(*t*) helps us achieve better test results compared to the SLRC model. Second, it also helps in reducing the number of neurons required in our LSNN model; for *d*-dimensional **x**(*t*), we require 2 × *d* neurons in the **ENC** layer, followed by 3 × *d* neurons in the **HDN** layer. Thus, the LSNN model requires a total of 5 × *d* number of spiking neurons. Considering the values of *d* in our LSNN experiments (refer Table 4), the minimum and maximum number of employed neurons in our LSNN model is 50 and 120, respectively, which is far below the number of neurons required in the SLRC model, where each ensemble has either 100 or 200 neurons, and the minimum number of ensembles is 6 (refer Table 2). In Table 5, we compared our spiking results with the current SoTA on 4 experimented datasets (Dey et al., 2022), where the authors have used LSMs as their spiking reservoir model. We note that the authors (Dey et al., 2022) use a minimum and maximum of 2500 and 5000 spiking neurons, respectively, in their reservoir, with additional 15 neurons in their *Gaussian Spike Encoder* layer; these numbers are significantly higher than ours in the LSNN model (more than 40x).

One may argue that the computational resource efficiency and the superior inference performance of the LSNN model perhaps comes at the cost of increased energy consumption, since there is no spiking reservoir in the LSNN model, rather a non-spiking LDN module to extract the temporal features. However, we note that the non-spiking LDN module offers minimal computation overhead due to the linear static matrix operations. The Loihi-1 (Davies et al., 2018) and Loihi-2 (Orchard et al., 2021) chips have 3 and 6 (respectively) number of embedded x86 processor cores—which can be efficiently used for non-spiking LDN preprocessing to extract the temporal features, before they are encoded to spikes (Davies et al., 2021). SpiNNaker-2 has MAC arrays which also offer the feasibility of inexpensive matrix operations in neuromorphic setting (Yan et al., 2021). We next analyze the energy consumption of the SLRC, LSNN, and LSNN_nspk_ models on CPU and Loihi-1. We did not do energy profiling on GPUs because previous works (Blouw et al., 2019; Patel et al., 2021) have shown that energy consumption on GPU is generally higher than that on Loihi for per sample inference. This is because the GPUs are optimized for parallel processing of data (and not online). Moreover, the application domain of our work is most well-suited to small IoT/Edge devices with sensors and batteries (GPUs are generally space consuming).

### 5.3. Discussion on energy consumption

Table 6 shows the energy consumption (in milli-Joules) of our proposed models, per sample, on Intel Core i5-5257U CPU and Nahuku32 board (built with Loihi-1 chips). Note that for energy consumption analysis, we did not train/evaluate the models on the CPU or Loihi-1 boards with the training/test data. We rather built our SLRC, LSNN, and LSNN_nspk_ models in Nengo with randomly generated matrices and executed them with Nengo simulator on CPU and with NengoLoihi simulator on Loihi-1. An input signal of 140 time-steps was randomly generated, and for all the analyzed models, we set *d* = 10. For the SLRC model, we use the hyper-parameters' values mentioned in the first row of the Table 3; we keep each model (and its hyper-parameters) unchanged while executing it on CPU and Loihi-1 (we ran each model for 10 times on both platforms). **Max**, **Min**, and **Mean** columns in Table 6 for each model denote the maximum, minimum, and mean ± std of the energy consumption measurements in mJ. As can be seen in the Table 6, LSNN on Loihi-1 reports the minimum energy consumption scores per input sample. Compared to LSNN_nspk_, on average (i.e., **Mean**), LSNN on Loihi-1 is 31.27 times more energy efficient. Compared to SLRC model too on Loihi-1, on average, LSNN reports lower energy consumption (87.30mJ vs. 81.65 mJ, respectively). It's interesting to note that for *d* = 10 and *N*_sn_ = 100, where the SLRC model has 1250 neurons and LSNN has meager 50 neurons, the gain in energy efficiency with LSNN on Loihi-1 isn't much. However, on CPU, we see that on average, LSNN is 2.34 times more energy efficient than SLRC—this small gain to an extent is explained by the disparity in the number of neurons. To investigate further, we built a simple spiking network to represent a scalar over time. The network consisted of an input node (outputting 1) connected to an ensemble of *N* neurons that was probed. For *N* = 100 and *N* = 4096 each, we ran the network for 10 times on Loihi-1, and measured the energy consumption for each run. We found that for *N* = 100 and *N* = 4, 096, the network consumed 59.94 ± 0.59 mJ and 63.46 ± 0.57 mJ on average, respectively. Therefore, we surmise that energy consumption on Loihi-1 does not increase dramatically with the number of neurons. Detailed energy consumption plots can be found in the Supplementary material.

**Table 6:** Comparison of energy consumption (per sample) in **milli-Joules** (mJ) of our spiking models—SLRC and LSNN model, and the non-spiking variant of LSNN, i.e., LSNN_nspk_ on CPU and Loihi-1.

**Platform**	**SLRC**			**LSNN**			**LSNN**_**nspk**_		
	**Max**	**Min**	**Mean**	**Max**	**Min**	**Mean**	**Max**	**Min**	**Mean**
**CPU**	7359.72	5262.13	6285.71 ± 803.61	3031.73	2189.45	2689.23 ± 248.30	2780.94	2319.15	2553.59 ± 163.20
**LOIHI-1**	91.94	85.06	87.30 ± 2.08	**90.91**	**73.40**	**81.65** ± 5.79	–	–	–

The bold values indicate the minimum energy consumption values.

Note that we did not do inference with the LSNN model on Loihi, because PyTorch does not support Loihi deployment; as well as, because of the cross-library (i.e., PyTorch and Nengo/NengoLoihi) challenges to port trained weights. However, Loihi-2 which has been recently released, supports three-factor learning rules (based on the surrogate gradient approach, Zenke and Ganguli, 2018), and SNNs on it can be trained/deployed by using Intel's Lava library (Intel, 2021); thus, the LSNN model is quiet well-suited for training and inference-mode deployment on Loihi-2. Next, we revisit the Table 5 and make a subtle remark with respect to the LSNN's results on the Ford-B dataset. We see that authors (Dey et al., 2022) achieve 64.32% accuracy on Ford-B with an LSM based model, where unlike the training data, the test samples are noisy. However, the LSNN model and its derivatives achieve far better accuracy on Ford-B (28.607% max improvement). This subtly hints toward the LDN (and subsequently the LSNN) being more robust to noise than LSMs—although, this needs to be properly investigated. Nonetheless, with respect to the LSNN's (and LSNN_nhdn_'s) overall inference performance in Table 5, compared to Dey et al. (2022), we see that the LSNN has a clear advantage over the popular LSMs—both in terms of accuracy and resource efficiency. One likely limitation of both SLRC and LSNN models could be the large set of hyper-parameters to tune (Tables 2, 4). In our LSNN experiments, although exhaustive with a wide range of hyper-parameters' values (and 3 runs each), we could not identify any conclusive trends between the individual hyper-parameters and test accuracy, other than, that test accuracy increases with the increase in order *d* of the LDN (which is expected). There were a few weak correlations suggesting higher values of neuron gain ρ, and higher values of θ for higher values of *d* being helpful to LSNN's performance. Also, lower values of learning rate, i.e., η < 0.05 demonstrated better fit.

## 6. Conclusion and future work

The literature around spiking TSC is not very rich; only a few popular spiking models exist, namely: the LSMs and the DFRs, and a few others (Dominguez-Morales et al., 2018; Fang et al., 2020; Gautam and Singh, 2020). There's a scarcity of spiking TSC models which are not only high performing but also resource efficient and neuromorphic hardware friendly. In this work, we presented two novel spiking models for the TSC task of univariate signals, along with their theoretical details and detailed empirical analysis. We first presented an entirely spiking, RC based model—the **SLRC** model, which was deployed on Loihi-1 for inference and also served as a precursor to our next improved model—the **LSNN** (if one counts its spiking derivative with no hidden layer—the LSNN_nhdn_ as another, then in total three spiking models were presented). We also did the energy consumption analysis of both our models on CPU and Loihi neuromorphic hardware, and observed that the LSNN model not only establishes a new SoTA spiking results on the experimented datasets, but also achieves the best energy efficiency (on average) on Loihi-1, among the compared models. We also found that the energy consumption gap between the spiking networks (on Loihi-1) with as much as 40 times the difference in the number of spiking neurons, is not much—although, this observation should be put to rigorous tests. However, in the context of our work, this leaves enough room to further introduce deeper layers, or more neurons in a layer, or increase the dimensionality *d* of the state-space vector in the LSNN model to improve its performance, without being at the risk of dramatically increasing its energy consumption on Loihi. To further establish the efficacy of our LSNN model, we intend to evaluate it on the entire set of the multi-class univariate TSC datasets, publicly available at the Time Series Classification website. We note that currently, our proposed spiking models are limited to univariate signals, we plan to address this limitation too in future. Another avenue which was looked over in this work, is the neuromorphic on-chip training of the spiking models; both, SLRC and LSNN models were trained off-chip. As stated in the previous section, SNNs can be trained on Loihi-2 with Lava using Three-Factor Rule based learning; this could be another promising direction to look into. In the end, the key takeaway of this work should be the LSNN model, which not only performs better compared to the LSM based models, but is also frugally resource efficient with minimum energy consumption on Loihi-1.

## Data availability statement

Publicly available datasets were analyzed in this study. The datasets can be found here: https://www.timeseriesclassification.com/.

## Author contributions

RG proposed, designed, and experimented with the models presented in this work, and wrote the manuscript. TS discussed the design of the models and provided valuable inputs to refine and improve them. RG, TS, and YY analyzed the results, with YY overseeing the development of the models and supervising this project. All authors reviewed the manuscript, contributed to the article, and approved the submitted version.

## Footnotes

^1^ http://www.timeseriesclassification.com/index.php
