# Supplementary material for: Reservoir based spiking models for univariate Time Series Classification
Source: Front Comput Neurosci. 2023 Jun 8;17:1148284. doi: 10.3389/fncom.2023.1148284 (PMC10285304; doi:10.3389/fncom.2023.1148284)
Supplement: Supplementary file 1 [file Data_Sheet_1.PDF]

# Supplementary Material

## 1 PROOF OF EQ. 10

Here we show the proof of obtaining Eq. 10 from Eq. 9 in the main paper. Following is the Eq. 9 :

$$\mathbf{X}(s) = \mathbf{H}(s)(A'\mathbf{X}(s) + B'\mathbf{U}(s)) \tag{S1}$$

where $\mathbf{H}(s) = \mathcal{L}[h(t)] = \frac{1}{1+s\tau}$. Below is the proof (we replace $\mathbf{H}(s)$ to obtain the following).

$$\mathbf{X}(s) = \frac{1}{1+s\tau}(A'\mathbf{X}(s) + B'\mathbf{U}(s)) \tag{S2a}$$

$$\implies \quad \mathbf{X}(s)(1+s\tau) = A'\mathbf{X}(s) + B'\mathbf{U}(s) \tag{S2b}$$

$$\implies \quad \mathbf{X}(s) + s\tau\mathbf{X}(s) = A'\mathbf{X}(s) + B'\mathbf{U}(s) \tag{S2c}$$

$$\implies \quad s\tau\mathbf{X}(s) = A'\mathbf{X}(s) - \mathbf{X}(s) + B'\mathbf{U}(s) \tag{S2d}$$

$$\implies \quad s\tau\mathbf{X}(s) = (A' - I)\mathbf{X}(s) + B'\mathbf{U}(s) \tag{S2e}$$

$$\implies \quad s\mathbf{X}(s) = \frac{1}{\tau}(A' - I)\mathbf{X}(s) + \frac{1}{\tau}B'\mathbf{U}(s) \tag{S2f}$$

## 2 ENERGY CONSUMPTION PLOTS

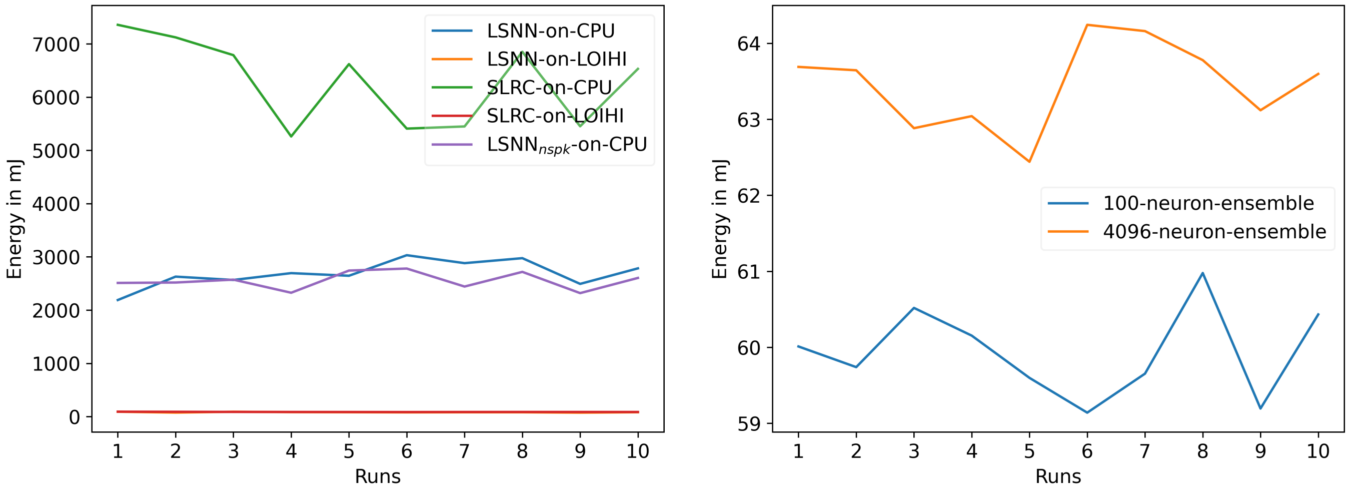


a. Plot for the energy consumption analysis of the SLRC, LSNN, and LSNN$_{\text{nspk}}$ models

b. Plot for the energy consumption analysis of the simple spiking network with $N = 100$ and $N = 4096$ neurons

**Figure S1.** Section 5.3 related energy consumption plots
